# A Comprehensive Literature Search of Digital Health Technology Use in Neurological Conditions: Review of Digital Tools to Promote Self-management and Support

**DOI:** 10.2196/31929

**Published:** 2022-07-28

**Authors:** John Henry Spreadbury, Alex Young, Christopher Myles Kipps

**Affiliations:** 1 Faculty of Medicine University of Southampton University Hospital Southampton NHS Foundation Trust Southampton United Kingdom; 2 Clinical Experimental Sciences Faculty of Medicine University of Southampton Southampton United Kingdom; 3 Wessex Neurological Centre University Hospital Southampton NHS Foundation Trust Southampton United Kingdom

**Keywords:** digital health technology, digital tools, neurology, patients, self-management

## Abstract

**Background:**

The use of digital health technology to promote and deliver postdiagnostic care in neurological conditions is becoming increasingly common. However, the range of digital tools available across different neurological conditions and how they facilitate self-management are unclear.

**Objective:**

This review aims to identify digital tools that promote self-management in neurological conditions and to investigate their underlying functionality and salient clinical outcomes.

**Methods:**

We conducted a search of 6 databases (ie, CINAHL, EMBASE, MEDLINE, PsycINFO, Web of Science, and the Cochrane Review) using free text and equivalent database-controlled vocabulary terms.

**Results:**

We identified 27 published articles reporting 17 self-management digital tools. Multiple sclerosis (MS) had the highest number of digital tools followed by epilepsy, stroke, and headache and migraine with a similar number, and then pain. The majority were aimed at patients with a minority for carers. There were 5 broad categories of functionality promoting self-management: (1) knowledge and understanding; (2) behavior modification; (3) self-management support; (4) facilitating communication; and (5) recording condition characteristics. Salient clinical outcomes included improvements in self-management, self-efficacy, coping, depression, and fatigue.

**Conclusions:**

There now exist numerous digital tools to support user self-management, yet relatively few are described in the literature. More research is needed to investigate their use, effectiveness, and sustainability, as well as how this interacts with increasing disability, and their integration within formal neurological care environments.

## Introduction

### Background

Neurological conditions present a human and economic challenge worldwide. How best to manage them remains a perennial issue. Digital health technology offers a potential solution. It would seem plausible that digital technology could play some role in supporting patients in self-management or health care professionals in the delivery of care. However, the digital health market contains a bewildering variety of websites, online platforms, and apps, some with empirical support, making it difficult to make sense of what is available, and their potential benefits. The objective of this paper was to conduct a literature search of the research on digital health technology in the self-management of neurological conditions, and to investigate what functions the technology provides and what benefits to users have been reported.

### Neurological Conditions

Neurological conditions refer to a group of medical disorders often resulting from disease or physical damage that affect the brain, or central or peripheral nervous systems. They can negatively impact patient mental health [1-4], psychological well-being [3], life satisfaction [3], health-related quality of life [5-8], cognitive functioning [3], and social support [9]. Worldwide, they are identified as significant predictors of disability and death [10,11]. They can also be detrimental to caregivers in terms of their mental health, quality of life, and caregiver burden [12-14].

As well as a human burden there is also an economic one. In the United Kingdom, statistics from the Neurological Alliance [15] indicate 16.5 million people in England have a neurological disorder. This statistic is equivalent to 1 in 6 of the population, and a prevalence believed to be increasing [15]. It is estimated that the National Health Service (NHS) cost of addressing neurological disorders is around £4.4 (US $3.0) billion [15], and may account for up to 14% of social care spending [5].

Many neurological conditions will be long term and incurable, and have symptoms that produce persistent or sporadic difficulties. Their onset may be sudden or gradual and their trajectories are marked by variance in stability or progression. Treatment and management may vary in complexity and include a combination of medication, rehabilitation, information, and support, and the involvement of a range of health care, allied health, and social care professionals [5].

Neurological conditions are generally managed in the community and there is increasing recognition of the importance of individuals self-managing their conditions [5,16]. Recent qualitative research by Kilinc et al [16] demonstrated the complex psychological and behavioral processes underlying self-management in neurological patients. The involvement of technology is one approach to supporting such efforts [5], while research by Gandy et al [3] indicated that there is interest among patients in web-based platforms to promote self-management.

### Digital Health Technology

Digital health technology, including terms such as eHealth, mobile health (mHealth), and digital tools, refers to the utilization, or application, of internet and smart-based technology to the promotion of health or health care [17]. Innovative technologies such as wearable devices, smartphone apps, internet-based self-help platforms, and health record databases have the ability to record, store, or present health-related data. This information can then be used to enhance the understanding, management, or monitoring of medical conditions by patients, carers, or health care professionals.

A range of digital technologies have already been applied to several individual neurological conditions such as epilepsy [18], MS [19], headache and migraine [20], Parkinson disease [21], and acquired brain injury [22]. There appears use and interest, at least in the short-term, and some evidence, with regard to web-based platforms, of a potential beneficial influence on mental health and quality of life [23]. However, it remains unclear how digital technologies become normalized within health behaviors and systems of care delivery in the medium-to-longer term. Furthermore, there may be significant patient and care-provider barriers that need to be considered [18,24,25].

A limitation of the present literature is that recent reviews and commentaries have tended to focus on individual neurological conditions (eg, [18-21,26-30]). There is an absence of reviews presenting digital tools across conditions that makes it difficult for clinicians and researchers, especially those new to digital health, to make comparisons, evaluations, and recommendations. It would be advantageous to know what digital tools are available to different patient groups, the underlying functionalities that support or promote self-management, and any salient psychosocial or clinical benefits for users identified.

### Aims

The literature search had several interrelated aims. First, we aimed to obtain an overview of the research on the use of digital health technology in the self-management of neurological conditions. Second, we aimed to identify the different types of digital health tools used by patients, carers, and health care professionals. Third, we aimed to develop an understanding of the underlying functionalities that allow digital health tools to support or promote self-management. Finally, we aimed to identify any salient outcomes, in terms of psychosocial or clinical benefits for users, associated with digital health technology use.

## Methods

### Literature Search Databases and Search Terms

We conducted a search of 6 databases: CINAHL, EMBASE, MEDLINE, PsycINFO, Web of Science, and the Cochrane Review. The searches were conducted using free text and equivalent database-controlled vocabulary terms. Search terms used were iteratively generated and informed by our interest in investigating digital health technology use in neurological conditions and neurodegenerative diseases. Multimedia Appendix 1 provides an example of the search terms.

Within each database, search terms were grouped into 2 categories: condition terms (ie, neurological conditions and neurodegenerative diseases) and digital technology terms. Search terms were combined using standard AND/OR commands. Where possible, filters were applied within databases to restrict searches to human participants, adults, and beginning from January 2000 onward. Searches spanned from January 2000 to February 2020, and were rerun in January 2021.

### Inclusion Criteria

The inclusion criteria for articles were as follows: Research conducted with human participants and published in English. Studies that had a focus on the use of digital health technology to help support self-management in patients or caregivers living with a neurological condition or neurodegenerative disease. Self-management was understood to refer to activities used to control a medical condition or maintain optimal health [31]. The self-management health component had to be delivered digitally, for example, via a computer, mobile/tablet app, or over the internet.

### Exclusion Criteria

Articles were excluded if they were not conducted with human participants or if they focused on artificial intelligence, biochemistry, computational modeling, diagnosis/assessment, cognitive stimulation/training, epidemiology, genetics, neuroimaging, neuropathology, physiotherapy, rehabilitation, scale development/validation, sensor technology, treatment, or interventions delivered by telephone. These areas were excluded to help narrow down the focus of digital health technology involved in self-management. Literature reviews, book chapters, study protocols, conference presentations, poster presentations, and unpublished theses were excluded.

### Search Methodology

Multimedia Appendix 2 shows that the overall search resulted in 26,572 articles being identified. Articles were downloaded into an Endnote library and duplicates were removed. The remaining articles were then exported to Rayyan reference management software, which allowed for the collaborative screening of articles by 2 reviewers. Articles were screened by reading the title and abstract of each article and applying the inclusion and exclusion criteria. Any articles where reviewers had conflicting opinions were discussed at the end of this process until consensus was met on inclusion or exclusion for full-text screening.

Following title and abstract screening, 96 articles moved forward to full-text screening. Microsoft Excel was used to list the 96 articles and extract salient information related to each article’s aims, methodology, results, and use of digital health technology. Full-text screening resulted in 45 articles being excluded for not meeting the inclusion criteria.

Rerunning the database searches and using keyword searches in Google Scholar resulted in 2 further articles being included. This resulted in a final total of 53 articles. A total of 27 articles focused on digital health technology use in neurological conditions and 26 on digital health technology use in dementia. The present paper only discusses the neurological condition articles.

### Study Methodological Quality and Value of Findings

We used the Critical Appraisal Skills Programme (CASP) Appraisal Tool to evaluate the methodological quality and value of the findings reported for each of the 27 included articles. All of the articles were considered to be of satisfactory methodological quality and produced findings of value. No cut-off scores were used and no articles were excluded as a result of using the tool.

### Analysis

The analysis is reported in 4 parts. First, we describe the contextual background of the articles. Using a data extraction table, we extracted from each article information about its nationality, the type of neurological condition studied, and methodological details (eg, participants, designs, outcome measures).

Second, we describe the digital health tools identified. From each article, we extracted information about the digital tool reported, including its name, the neurological condition it addressed, the format of the technology, its users, and its broad aims.

Third, we describe the underlying functionalities of the digital health tools that appeared to promote or support self-management. This information was obtained by extracting from each article the description of how each digital tool functioned. By iteratively reading through descriptions several different categories of function could be identified across the articles. These categories were then grouped together based on the similarity of functions to create 5 overarching categories that represented the main functionalities provided by the digital tools.

Finally, we describe salient psychosocial and clinical benefits associated with the digital health tools. This information was obtained by extracting the main outcomes reported that reflected psychosocial or clinical benefits to users.

Preliminary data analysis, findings, and interpretations from the review were presented at internal research group meetings for sense checking and feedback.

## Results

### Contextual Background

The search identified 27 articles. These articles came from 9 different countries. The majority of articles were from the United States with 15. This was followed by 4 articles from Holland and 2 from Australia. There was 1 article each from Belgium, Germany, New Zealand, and Turkey. One additional article reported on a sample including participants from the UK and Canada, and 1 with participants from the UK and New Zealand.

A total of 10 articles focused on MS, 6 on epilepsy, 6 on stroke, 4 on headache or migraine, and 1 on pain. Two articles with a focus on MS also included participants with Parkinson disease and postpolio syndrome. The majority of articles centered on patients (n=21), with a minority on carers (n=4). One article included patients and carers, and 1 patients and health care professionals.

The majority of articles reported studies using quantitative or mixed quantitative-qualitative designs. Only 2 articles reported qualitative studies. Across the articles a range of measurements were employed, including widely used questionnaire instruments (eg, on mental health, fatigue), process evaluation metrics (eg, usability, satisfaction), digital technology system metrics or stored data (eg, recorded usage of a digital tool), and open-ended questions (eg, on subjective experience).

We identified approximately 100 questionnaire instruments, including instruments used more than once. When these instruments were broadly grouped together based on the similarity of construct being measured, 16 measurement domains could be identified (Table 1). Among the most prevalent areas measured were mental health, quality of life, fatigue/physical activity, disability, and self-efficacy.

**Table 1:** Estimate of measurement domains by percentage of questionnaire instruments used.

Measurement domain	%
Mental health	16
Quality of life/life satisfaction	13
Fatigue/activity	10
Disability	10
Self-efficacy	9
Coping/control	9
Self-management	6
Stress	4
Usability	4
Sleep quality	3
Social support	3
Care satisfaction/quality	3
Health care utilization	3
Improvement	2
Health status	2
Condition knowledge	2
Other	3

### Digital Tools and Aims

Table 2 shows that 17 different digital tools were reported across the articles. A number of them, for example, PatientsLikeMe, WebEase, Mymigraine, and Caring-Web, were reported by more than 1 article. The majority of digital tools were website/web-based platforms and a minority were smartphone apps.

MS had the highest number of reported digital tools with 8, and this was followed by epilepsy and stroke both with 3, and headache and migraine with 2. The platform painACTION was reported in 2 different conditions—headache and migraine, and pain. The majority of digital tools focused on patients, while only 2 platforms, both related to stroke, focused on carers.

In the MS group, there were tools that specifically targeted fatigue and depression as well as personal health record management. In epilepsy, there were tools that involved collaborative self-management with a health care professional and information sharing within a health-related social network. For stroke, provision of stroke-related education was offered to carers and patients. In headache and migraine, tools provided training to promote self-management potential, and in pain there was a digital tool that addressed cognitive and emotional aspects of pain self-management.

**Table 2:** Digital health technology by neurological condition, type of technology, users, and aim.

Condition and digital technology name		Type of technology	Users	Broad aim
**Multiple sclerosis**				
	MS Energize	Smartphone app	Patients	Fatigue self-management
	Problem Solving Therapy	Website/web-based platform	Patients	Depression self-management
	MS TeleCoach	Smartphone app	Patients	Physical activity/fatigue self-management
	Deprexis	Website/web-based platform	Patients	Treatment of depression
	MSdialog	Web-based/smartphone app	Patients	Multiple sclerosis management/health data sharing
	Mellen Center Care Online	Website/web-based platform	Patients	Personal health (record) management/self-management
	PatientSite	Website/web-based platform	Patients/health care professionals	Personal health (record) management system
	MSInvigor8	Website/web-based platform	Patients	Self-management/fatigue self-management
**Epilepsy**				
	MINDSET	Tablet-based platform	Patients/health care professionals	Shared clinical decision tool/self-management
	PatientsLikeMe^a^	Website/web-based platform	Patients	Data sharing/health social network/understanding
	WebEase^a^	Website/web-based platform	Patients	Epilepsy self-management
**Stroke**				
	Stroke Carer Support	Website/web-based platform	Carers	Carer education/enhance understanding/capability
	Caring Web^a^	Website/web-based platform	Carers	Carer education/support
	Post-Discharge Support	Website/web-based platform	Patients	Education/information provision/coping
**Headache/migraine**				
	painACTION^a^	Website/web-based platform	Patients	Migraine self-management/coping/self-efficacy
	Mymigraine^a^	Website/web-based platform	Patients	Behavior training/self-management
**Pain**				
	painACTION^a^	Website/web-based platform	Patients	Pain self-management
**Multiple sclerosis, Parkinson disease, postpolio syndrome**				
	Fatigue Self-Management Program^a^	Website/web-based platform	Patients	Fatigue self-management

^a^Reported in more than 1 article.

### Digital Tools and Functionality

We identified 5 broad categories of interrelated functionality across digital tools: (1) knowledge and understanding; (2) behavior modification; (3) self-management support; (4) facilitating communication; and (5) recording condition characteristics.

### Knowledge and Understanding

Around two-thirds of the digital tools had functionality involving increasing neurological condition knowledge and understanding. This category included tools providing psychoeducational/​self-help information and cognitive behavior therapy guidance. Users could engage with learning-orientated “modules” or “lessons,” often presented using interactive multimedia formats, and in some cases the completion of “homework” activities [32-38].

Around half of the digital tools provided some form of psychoeducational/self-help information. This support could include information on medical or psychosocial issues, coping and managing, or healthy living, and in some cases internet links to related resources [32,33,35,38-41]. In the case of stroke carers, there was comprehensive information on caring for a patient with stroke at home [41,42].

Approximately one-third of digital tools drew on or included a cognitive behavior therapy component. This function involved engagement with learning activities that encouraged users to address challenging condition–related cognitions, behaviors, lifestyles, or expectations; increase self-awareness or self-understanding; and learn new skills and their application [23,32-34,36,38,43,44].

### Behavior Modification

Around one-third of digital tools aimed to prompt behavior modification and included a focus on stimulating behavior change and providing coaching or motivation. A small number of tools addressed behavior change using activities such as assessment and evaluation of behavior, establishing behavior objectives, and utilizing “action plans” [39,45-47]. Selected digital tools also had the ability to provide user feedback, “motivational” messaging, advice, reminders, or encouragement [35,39,48-50].

### Self-management Support

Overlapping with behavior modification were digital tools with the function of facilitating users in psychological or tangible self-management. This function assisted users in contemplating their own or preferable self-management, in some cases bolstered by feedback, and encouraged consideration of processes or targets to aid enhancement [39,45-47,49]. Tangible self-management was offered by the PatientSite platform that permitted users to access aspects of their own health record including their medical record, test results, health care appointments, and medication prescriptions [40].

### Facilitating Communication

Approximately half of the digital tools facilitated communication either between users and health care professionals or peer-to-peer. Communication was often asynchronous, could be condition or intervention related, and used various formats, for example, email or discussion groups [38,39,44,46]. User communication with health care professionals could involve sharing health information, making requests, or asking questions [40,41,51], while health care professional communication could take the form of replies to users, supportive messages, reminders, or feedback [35,38,44]. Peer-to-peer communication could involve sharing experiences or advice [7,35,39].

### Recording Condition Characteristics

Around one-third of digital tools included a function for recording condition-related information that could then be “tracked,” “monitored,” or “shared” to enhance management or understanding [7,34,39,45,51,52]. Finally, there was a digital tool, Caring Web, that included an entertainment function, whereby users had access to amusements (eg, “jokes” and “games”) and topical news features [41].

### Digital Tools and Outcomes

For the majority of digital tools some form of acceptability (eg, effectiveness, feasibility) was reported. This could be in the context of user responses, as a method of data collection, or in producing certain outcomes.

Self-management per se was seldom measured but instead proxies were used such as self-efficacy or coping. Where condition self-management could be directly measured as in epilepsy, digital tools such as WebEase and PatientsLikeMe were associated with enhanced self-management [39,52]. Across the conditions migraine, epilepsy, and a sample including MS, Parkinson disease, and postpolio syndrome, the digital tools painACTION, WebEase, Fatigue Self-Management, PatientsLikeMe, and Mymigraine were associated with improved condition-related self-efficacy [33,35,36,39,44,46,52]. Across the conditions migraine, pain, and stroke, the digital tools Mymigraine, painACTION, and Post-Discharge Support, respectively, were associated with either increased coping or use of positive coping strategies [33,34,37,50].

Depression was a frequently measured outcome and produced mixed findings. Scales used to measure depression included the Beck Depression Inventory [53]; Depression, Anxiety, and Stress Scale [54]; Hospital Anxiety and Depression Scale [55]; and the Centre for Epidemiological Studies Depression scale [56]. Across the conditions MS, migraine, and pain, digital tools such as Problem Solving Therapy, painACTION, Deprexis, and Fatigue Self-Management were associated with lower depression [23,33-35,43]. However, across the conditions MS and stroke, digital tools such as MS TeleCoach, Fatigue Self-Management, MSInvigor8, and Caring-Web showed no association with depression [38,48,57,58].

An outcome frequently measured in MS articles was fatigue and robust findings were identified. Measures of fatigue included the Fatigue Scale for Motor and Cognitive Functions [59] and a version of the Fatigue Impact Scale [60]. Digital tools such as MS TeleCoach, Deprexis, Fatigue Self-Management, and MSInvigor8 were associated with better fatigue scores [23,35,38,48,57]. Although quality of life was frequently measured, only the digital tool Deprexis appeared to show a positive influence [23].

## Discussion

### Principal Findings

This review provides an overview of self-management digital tools across a number of neurological conditions. The findings offer a complementary perspective to the literature on digital tool development and implementation by focusing on functionality and beneficial outcomes. Five broad categories of interrelated functions can be discerned that allow digital tools to promote self-management. Among these functions are the provision of information to increase knowledge and understanding; encouragement of positive behavior change; support in psychological and tangible self-management; facilitating communication between users and health care professionals or users in a similar situation; and the ability to record, monitor, and share condition information.

The digital tools appeared modestly associated with psychosocial or clinical benefits to users. Depression was frequently measured and yet while some digital tools indicated potential for reducing depression, for others there was no association. By contrast, a number of MS digital tools demonstrated some potential in managing fatigue. Interestingly, self-management in itself was seldom measured outside of epilepsy; however, certain digital tools were associated with increased self-efficacy and use of positive coping strategies.

Across the literature we found little discussion about health service adoption or endorsement of digital tools or how they fit with the formal neurological care individuals receive [32,35]. For health service adoption, functionalities and user outcomes should be compatible with existing models of care. Functionalities such as promoting knowledge and understanding, facilitating communication with health care professionals, and recording condition information may lend themselves well to health service adoption. However, the evidence of user benefits may still be too limited. Indeed, future research should test digital tools by embedding and evaluating them within clinical care pathways. As such, the digital tools reviewed may best be considered as supplementary resources to any formal neurological care being received.

There was also little discussion across the literature about uptake and continued use of digital tools beyond a research context [38,39]. As part of analyzing articles, using the internet to conduct searches, we found it difficult to identify whether some digital tools were still in use or not. Indeed, future research could attempt to establish how many of the digital tools reported are still in use and how many have been abandoned and why (eg, changes in technology, low user uptake, cost).

There are a number of methodological limitations that should be considered. We excluded articles focused on assessment, cognitive training, physiotherapy, and sensor technology and this could have influenced the findings. These articles were excluded as at an early stage of screening it was judged that these areas contributed more to diagnosis, rehabilitation, and assistive technology than self-management. We did not identify as many self-management apps as we had expected; this may have been caused by not including within our searches the brand names of any apps or app marketplaces; however, more likely, many apps exist that are simply not reported in the scientific literature. Furthermore, we did not search the gray literature for self-management apps.

Future research should try to establish user preferences toward identifying the functions used most frequently, considered most useful, and that produce clinical benefits. Research should also consider whether user needs and preferences are being addressed. Prospective research could investigate the effect of medium-to-longer-term usage on user outcomes, and the effect on formal neurological care usage. Understanding the effect of integrating data from digital tools into formal clinical records, and the impact of utilizing multiple different tools simultaneously would also be worthwhile.

### Conclusions

Digital health technology has been applied to a number of neurological conditions, yet there is a relatively limited literature on its use and usefulness in the context of self-management. It is likely that numerous other apps and websites have yet to enter the research literature. Detailed analysis and description of the self-management process is lacking as are condition-specific self-management scales, comparison of digital tools, and consideration of comparative outcomes. There appear to be modest associations with psychosocial or clinical outcomes but evaluation is needed of whether certain functionalities predict certain outcomes.

## Appendix

Multimedia Appendix 1 Example of search terms.

Multimedia Appendix 2 Flowchart of the literature search.

## Abbreviations

CASP: Critical Appraisal Skills Programme
mHealth: mobile health
MS: multiple sclerosis
NHS: National Health Service
