# Supplementary material for: A Comprehensive Literature Search of Digital Health Technology Use in Neurological Conditions: Review of Digital Tools to Promote Self-management and Support
Source: J Med Internet Res. 2022 Jul 28;24(7):e31929. doi: 10.2196/31929 (PMC9377435; doi:10.2196/31929)
Supplement: Multimedia Appendix 1 [file jmir_v24i7e31929_app1.docx]

**Condition**

Neurology and Neurological Conditions

- Epilepsy
- Seizures
- Multiple Sclerosis
- Neuroinflammatory
- Parkinson’s Disease
- Movement disorders
- Motor Neurone Disease
- Neuro-muscular
- Headache
- Migraine

Dementias and Neurodegenerative Diseases

- Alzheimer’s Disease
- Vascular Dementia
- Dementia with Lewy Bodies
- Presenile Dementia
- Posterior Cortical Atrophy
- Huntington's Disease
- Frontotemporal Dementia (FTD) or Frontotemporal Lobar Degeneration (FTLD)
- Pick’s Disease or Pick Complex
- Behavioural-Variant Frontotemporal Dementia (bvFTD)
- Semantic Dementia (SD)
- Progressive Non-Fluent Aphasia (PNFA)
- Primary Progressive Aphasia (PPA)
- Progressive Supranuclear Palsy (PSP)
- Corticobasal Degeneration (CBD)
- Prion Disease
- Mild Cognitive Impairment (MCI)
- Subjective Cognitive Impairment

**Digital Health Technology**

Digital healthcare technologies

- Electronic health (E-health. mhealth)
- Digital health interventions
- Digital health
- Smart technology
- Digital apps (applications)
- Smart devices
- Patient held record
- Digital/Online care planning
- Online
- Virtual
- Web-based
