# Supplementary figures and images for: A Comprehensive Literature Search of Digital Health Technology Use in Neurological Conditions: Review of Digital Tools to Promote Self-management and Support
Source: J Med Internet Res. 2022 Jul 28;24(7):e31929. doi: 10.2196/31929 (PMC9377435; doi:10.2196/31929)

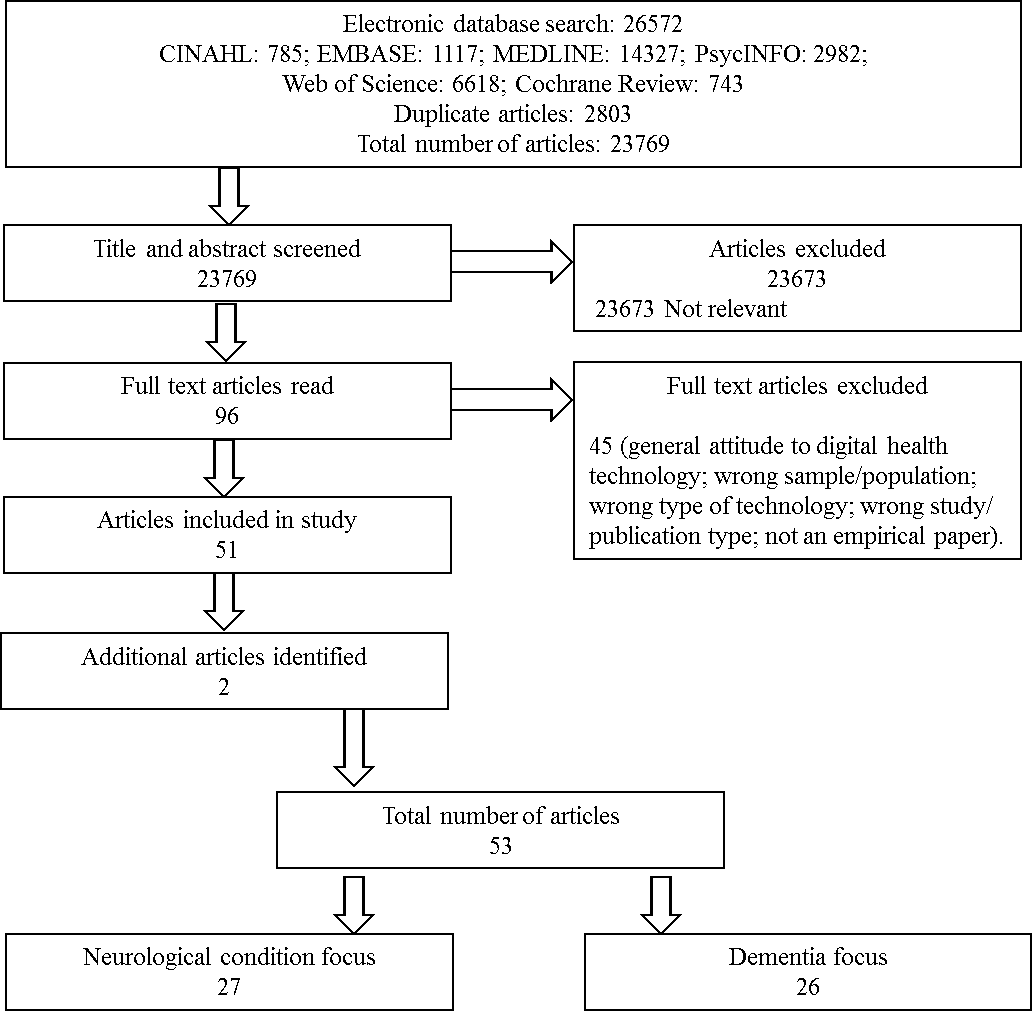

Supplement: Multimedia Appendix 2 [file jmir_v24i7e31929_app2.png]
